# P01-03 Movement and physical activity in early childhood education and care in the Nordic countries

**DOI:** 10.1093/eurpub/ckac095.003

**Published:** 2022-08-29

**Authors:** Ann-Christin Sollerhed, Line Grønholt Olesen, Anne Soini, Arja Sääkslahti, Gudrún Kristjánsdóttir, Rúnar Vilhjálmsson, Ingunn Fjørtoft, Robert Larsen, Jan-Eric Ekberg, Karsten Froberg

**Affiliations:** 1 Kristianstad University, Kristianstad, Sweden; 2 University of Southern Denmark, Odense, Denmark, Odense, Denmark; 3 University of Jyväskylä, Finland, Jyväskylä, Finland; 4 University of Iceland, Reyjkavik, Iceland, Reykjavik, Iceland; 5 University of South-Eastern Norway, Notodden, Norway; 6 Malmö University, Malmö, Sweden

**Keywords:** curriculum, movement, physical activity, early childhood education and care, children

## Abstract

**Background:**

The World Health Organization (WHO) acknowledges the importance of preschool children taking part in comprehensive physical activities supporting, among other things, their motor development, and competencies. A growing number of children attend early childhood education and care (ECEC), and expectations that this will support the development and learning of the youngest children are high. ECEC are governed by different policies embodied in both laws and curricula, and the framework of a curriculum plays a key role in ensuring the quality of ECEC services. The documents represent the content society wants the ECEC institutions to disseminate, and set out the values, objectives, and content of the work of pre-school teachers and serve as a point of reference for ECEC teachers and schools. The purpose of this study was to examine the values of movement and physical activity (MoPA) using government policy documents ECEC from Denmark, Finland, Iceland, Norway, and Sweden.

**Methods:**

This descriptive, comparative study was designed based on curriculum theory and used word count and content analyses to examine values of MoPA and to identify similarities and differences in the ECEC policies of Nordic countries.

**Results:**

Seven terms were identified as MoPA related; body, motor, move, physical activity, physical education, coordination, idrott/liikunta. These terms occurred in various content contexts: development, environment, expression, health and well-being, learning and play, albeit sparsely and were referred to as both a goal in itself and as a mean of achieving other goals (e.g., learning or development in another area). Formulations dedicated to MoPA as a goal were present in the Danish and Finnish curricula and, to some extent, also in the Norwegian, while the Icelandic and Swedish curricula mentioned MoPA only as a mean.

**Conclusion:**

Findings indicated that MoPA, which are important for children's development, health, and well-being, is a low-priority value, to varying degrees, in the ECEC policies enacted by the Nordic countries. Thus, the guidance provided to educators and stakeholders therein is inexplicit. The low priority of the MoPA domain in the ECEC policies might negatively affect the possibility for young children to be physically active in preschools.

